# COVID-19 Mortality and Remdesivir – A Retrospective Cohort in Intensive Care Setting

**DOI:** 10.7759/cureus.51002

**Published:** 2023-12-23

**Authors:** Elizabeth S Xavier, Vishnu R Nair, Shahanas P Shajahan, Abdul Raheem, Geetha Philips, Praveen Valsalan, Manu Pradeep

**Affiliations:** 1 Pulmonology, Aster Medcity, Kochi, IND; 2 Nephrology, Sheffield Teaching Hospitals NHS Foundation Trust, Sheffield, GBR; 3 Internal Medicine, Aster Medcity, Kochi, IND; 4 Department of Non-Communicable Disease Epidemiology, London School of Hygiene & Tropical Medicine, London, GBR

**Keywords:** icu mortality, clinical effectiveness, remdesivir, critical care, covid-19

## Abstract

Background: Remdesivir is a broad-spectrum antiviral drug that received emergency use authorization in the first wave of the COVID-19 pandemic. However, its effectiveness in preventing mortality in COVID-19 patients who required intensive care was unclear.

Patients and methods: We retrospectively analyzed clinical data of 302 patients from intensive care units of a quaternary care center with moderate to severe COVID-19 illness and followed them until discharge between March 2020 and February 2021. Participants who received at least five doses of Remdesivir were compared against participants who received standard care. The primary outcome was all-cause mortality. Secondary outcomes included invasive mechanical ventilation, clinical worsening, and intensive care stay.

Results: Remdesivir use was not associated with all-cause mortality in this cohort (age and sex-adjusted OR = 0.76, 95% CI 0.4 -1.5, p = 0.409). However, when stratified for clinical severity and steroid use, Remdesivir demonstrated a strong negative association with all-cause mortality in severely ill patients (OR 0.3, 95% CI 0.1 - 0.6, p = 0.003) or when used along with intravenous Methylprednisolone (Infusion/Bolus, OR 0.2/0.3, 95% CI 0.1 - 0.9 p = 0.06). Remdesivir use was not significantly associated with invasive mechanical ventilation or clinical worsening but with prolonged ICU stay.

Conclusion: While Remdesivir use may not affect all-cause mortality in moderate to severely ill COVID-19 ICU patients, it may still benefit severely ill patients or when used with intravenous steroids. However, the limitations of the present study necessitate a randomized controlled trial to test this combined intervention strategy.

## Introduction

Early in the COVID-19 pandemic, there were sparse treatment options, and most guidelines relied on empirical evidence from other viral respiratory infection outbreaks like Severe Acute Respiratory Syndrome (SARS) and Middle-Eastern Respiratory Syndrome (MERS) [1]. The first wave of infections, estimated to have extended between January 2020 and February 2021, is estimated to have infected about 35% of the 1.3 billion Indian population, with a case-fatality ratio of 5 to 15% [2].

Anticipating a dire scenario in India - in June 2020, Remdesivir received emergency use authorization from the Central Drugs Standard Control Organization (CDSCO) in India and was included as an investigational therapy under the National Clinical Management Protocol (NCMP) for moderate and severe COVID-19 illness by consensus of an expert committee [3,4]. Remdesivir (GS-5734) is a broad-spectrum antiviral that targets replication in pathogenic ribonucleic acid (RNA) viruses, including MERS and SARS viruses, initially shown to be efficacious in in-vitro settings and animal models [5-7]. The World Health Organization had already voiced skepticism about its real-world effectiveness in reducing mortality in COVID-19 infections at the time [8]. Regardless of the uncertainty, Remdesivir was used by selected participating centers in India during the first wave as an investigational drug, which was actively surveilled for clinical and safety outcomes [4].

In this retrospective cohort study, our primary objective is to investigate the association of Remdesivir use with all-cause mortality before hospital discharge in moderate to severe COVID-19 patients who required intensive care during the first wave of the pandemic. As our secondary objectives, we sought to evaluate the association between Remdesivir use with invasive mechanical ventilation/extra-corporeal membrane oxygenation and worsening disease severity. We believe that this analysis would add to the real-world evidence surrounding the uncertain effectiveness of Remdesivir for its use in moderate to severe COVID-19 patients [9].

## Materials and methods

Study design, settings, and participants

The current study adopted a retrospective cohort design and used data from a more extensive active surveillance program that collected clinical and safety outcomes in all COVID-19 patients admitted to the participating center between 1st March 2020 and 28th February 2021 (12 months). The institutional ethics committee approved the study (AM/EC/324-2023). The informed consent requirement was waived as it was a retrospective study using routinely collected clinical data. Participant data were sourced from the Intensive Care Unit (ICU) records of Aster Medcity, a Joint Commission International accredited quaternary care hospital in Kochi, India. Participants were included in the current study if they had been admitted to the ICU with moderate to severe COVID-19 pneumonia, as per the NCMP protocol at the time (Table 1) [3]. In-house laboratory confirmation of a SARS-COV-2 infection was obtained by either Reverse Transcriptase - Polymerase Chain Reaction (RT-PCR) tests or a Rapid Antigen Test (RAT). Participants were excluded from the study if they were under 18.

**Table 1:** Clinical severity classification as per National Clinical Management Protocol SpO_2_ - Blood Oxygen saturation.

Clinical Severity	Clinical Presentation	Clinical Parameters
Mild	Patients with uncomplicated upper respiratory tract infection - may have mild symptoms such as fever, cough, sore throat, nasal congestion, malaise, and headache.	Adults without shortness of breath or hypoxia (SpO_2 _≥ 94% on room air).
Moderate	Pneumonia with no signs of severe disease	Adults with clinical features of dyspnea or hypoxia, fever, cough, SpO_2 _≤ 90 to 93% on room air, and respiratory rate ≥ 24 per minute.
Severe	Severe Pneumonia	Adults with clinical signs of pneumonia plus one of the following; respiratory rate > 30 breaths/minute, severe respiratory distress or SpO_2 _< 90% on room air. Adults with Acute Respiratory Distress Syndrome or sepsis or septic shock.

Intervention groups and outcomes

All patients received standard care of a quaternary ICU facility, including mechanical (respiratory support, bronchoalveolar lavage, chest physiotherapy) and pharmacological interventions (steroids, antibiotics, anticoagulants, diuretics), as per the NCMP guidelines [3]. When steroid use was indicated as part of standard care, multimorbid diabetic patients with poorly controlled blood glucose levels received Inj. Methylprednisolone 80mg as a continuous infusion (along with insulin), while the others received Inj. Methylprednisolone 40mg 12th hourly as bolus doses. Both regimens were titrated according to clinical response for 5 to 10 days.

The present study primarily focused on the clinical and safety outcomes of the intravenous use of Inj. Remdesivir 100mg/20ml (Cipremi®; Cipla Ltd, Mumbai, India), when used in moderate to severe COVID-19 pneumonia patients admitted in the ICU. Participants were classified as the "Remdesivir" group when they had received at least five or more doses of the drug in the same setting. Other participants admitted to the unit before the drug's approval by CDSCO were classified as the "Control" group. Patients who died or developed a derangement in their liver functions (transaminase elevation three times the normal upper limit, and serum bilirubin two times the normal upper limit - requiring cessation of the drug) before the standard five-day regimen was completed - were also included in the "Control" group.

The primary clinical outcome was the in-hospital all-cause mortality of the included participants. Secondary clinical outcomes included the proportion of participants who required invasive mechanical ventilation or extracorporeal membrane oxygenation support and an escalation in illness category (moderate to severe - as per NCMP protocol) after admission to the ICU. Safety outcomes included clinically diagnosed bleeding complications, acute kidney injury, and antibiotic escalation requirements in the participants. As a sub-group analysis amongst the "Remdesivir" group, the Symptom Onset to Remdesivir Treatment (SORT) interval was calculated and assessed for its association with all-cause mortality [10].

Data were abstracted by trained clinical researchers using bespoke clinical research forms from the participant's clinical notes and electronic health records. The investigators ensured that the participant's anonymity was preserved through reversible coding during data entry. An independent external researcher validated the data quality and performed the data analysis.

Statistical analysis

First, an exploratory analysis was conducted. The baseline demographic characteristics, clinical profile at admission, and various therapeutics received were summarised using frequencies and percentages for categorical variables. Continuous variables were summarised with mean and standard deviation. After recoding into dummy variables, their associations with all-cause mortality were tested using univariate logistic regression.

Second, a causal model of regression analysis was attempted. The demographic, clinical, and therapeutic variables distribution amongst the "Remdesivir" vs "Control" group was assessed. Pearson's Chi-square test or Fischer's Exact test was used when data were categorical, and independent t-test when the data was continuous. Next, a crude estimate of the association between Remdesivir use and all-cause mortality was derived using a univariate logistic regression. When individually adjusted in a bivariate logistic regression, other exposure variables were deemed confounders if they caused a change in the crude estimate. They were further screened for effect modification using a likelihood ratio test. Exposure variables that demonstrated multicollinearity with other confounders were excluded from the final model. Next, confounders were added stepwise to the final multivariate logistic regression model to derive an adjusted estimate of association. This adjusted estimate was then further stratified by the effect modifiers.

Estimates were reported as Odds ratio (OR), 95% confidence intervals (95% CI), and p values. A two-sided p-value of 0.05 was determined to be statistically significant. All statistical analyses were performed on StataSE Version 17 (StataCorp LLC, Texas, USA) and SPSS for Windows - Version 28 (IBM Corp. Armonk, NY, USA).

## Results

In this retrospective cohort analysis of the 305 patients admitted with COVID-19 illness in the ICU, 302 patients met the eligibility criteria (three patients were under 18 years of age) and were included in the analysis. There were no missing data. In an exploratory analysis (Table 2), a plurality of participants were over 65 years old (n =112, 37.1%) and were strongly associated with all-cause mortality.

**Table 2:** Cohort characteristics at admission and their univariate associations to all-cause mortality (N=302) HRCT – High Resolution Computed Tomography, *n = 3, HRCT not taken

Baseline characteristics	Deaths/Total (%)	Odds Ratio	95% C.I	p-value
Age in years				
18-34	1/26 (3.8%)	(Reference)	-	-
35-54	15/81 (18.5%)	5.7	0.7 – 45.3	0.1
55-64	9/83 (10.8%)	3.0	0.4 - 25	0.3
65+	29/112 (25.9%)	8.7	1.1 - 67.4	0.038
Sex				
Male	47/225 (20.9%)	2.6	1.1 - 6.1	0.024
Female	7/77 (9.1%)	(Reference)	-	-
Comorbidities				
Diabetes Mellitus	29/161 (18%)	1.0	0.6 - 1.8	0.949
Hypertension	29/151 (19.2%)	1.2	0.7 - 2.2	0.548
Chronic Liver Disease	4/12 (33.3%)	2.4	0.7-8.3	0.166
Chronic Kidney Disease	8/36 (22.2%)	1.4	0.6 – 3.2	0.47
Ischaemic Heart Disease	18/78 (23.1%)	1.6	0.8 - 2.9	0.167
Chronic Lung Disease	4/23 (17.4%)	1.0	0.3 – 3.2	0.949
Chronic Neurological Disease	1/8 (12.5%)	0.7	0.1 – 5.4	0.650
Stroke	3/17 (17.6%)	0.9	0.3 – 3.5	0.979
Pregnancy	0/11 (0%)	0	-	-
Cancer	7/21 (33.3%)	2.5	0.9 – 6.5	0.063
Immunocompromised	0/3 (0%)	0	-	-
Morbidity count				
0-1	24/139 (17.3%)	(Reference)		
2-3	22/141 (15.6%)	0.9	0.5-1.7	0.707
> 3	8/22 (36.4%)	2.7	1-7.2	0.043
Admission Category				
Moderate	10/114 (8.8%)	(Reference)		
Severe	44/188 (23.4%)	3.2	1.5–6.6	0.002
Category escalation				
No change	46/256 (17.9%)	(Reference)		
Moderate to Severe	8/46 (17.4%)	0.9	0.4-2.17	0.898
Admission HRCT Scoring				
Normal*	22/120 (18.3%)	(Reference)		
Mild	4/41 (9.8%)	0.5	0.2-1.5	0.205
Moderate	7/60 (11.7%)	0.6	0.2-1.5	0.255
Severe	21/81 (25.9%)	1.6	0.8-3.1	0.200

Most participants were male (n = 225, 74.5%) and strongly associated with all-cause mortality. Most participants (n = 163, 53.9%) had two or more comorbidities, with Diabetes Mellitus and Systemic Hypertension being the two most prevalent morbidity in the cohort. Except for "Cancer", no other comorbidity was associated with all-cause mortality (Table 2). Furthermore, most participants were admitted with severe COVID-19 disease (n =188, 62.2%), and a large proportion required mechanical ventilation (n = 72, 23.8%). These participants demonstrated a strong association with mortality (Table 3). Two hundred and four participants (67.5%) received at least five doses of Remdesivir, and 56 participants (18.5%) died in the cohort prior to hospital discharge.

**Table 3:** Co-interventions, complications, and their associations to all-cause mortality (N=302) LMWH: Low Molecular Weight Heparin; HFNC: High Flow Nasal Cannula, BiPAP: Bilevel Positive Airway Pressure, ECMO: Extracorporeal membrane oxygenation; # - Teicoplanin, Vancomycin, Clindamycin, Levofloxacin

Variables	Deaths/Total (%)	Odds Ratio	95% C.I	p-value
Initial mode of ventilation				
Room air	9/95 (9.5%)	(Reference)		
Face mask	8/60 (13.3%)	1.5	0.5-4	0.456
Nasal prongs	8/73 (11%)	1.2	0.4-3.2	0.752
HFNC	1/2 (50%)	9.5	0.5-166.1	0.121
BiPAP	18/51 (35.3%)	5.2	2.1-12.6	<0.001
Invasive Mechanical Ventilation	10/21 (47.6%)	8.7	2.9-26	<0.001
Escalation in ventilation				
No escalation	13/181 (7.2%)	(Reference)		
Face mask/Nasal prongs/HFNC	0/18 (0%)	-	-	-
BiPAP	2/50 (4%)	0.5	0.1-2.5	0.426
Invasive Mechanical Ventilation	38/50 (76%)	40.9	17.3-96.7	<0.001
ECMO	1/3 (33.3%)	6.5	0.5-76	0.138
Steroid administration				
None	4/67 (6%)	(Reference)		
Bolus	26/146 (17.8%)	3.4	1.1-10.2	0.028
Infusion	24/89 (27%)	5.8	1.9-17.7	0.002
Steroid Dose				
None	4/66 (6.1%)	(Reference)		
Methylprednisolone 40mg	15/76 (19.7%)	3.8	1.2-12.1	0.024
Methylprednisolone 80mg	35/160 (21.9%)	4.3	1.5-12.8	0.008
Antibiotics used				
None	4/49 (8.2%)	(Reference)		
Augmentin/Azithromycin	2/37(5.4%)	0.6	0.1-3.7	0.621
Cefperazone	12/93 (12.9%)	1.7	0.5 - 5.5	0.4
Meropenem	19/35(54.3%)	13.4	4 - 45.2	<0.001
Piperacillin + Tazobactam	14/81 (17.3%)	2.4	0.7-7.6	0.153
Others#	3/7 (42.9%)	8.4	1.4-51.7	0.021
Antibiotic escalation				
None	24/224 (10.7%)	(Reference)		
Escalated	30/78 (38.5%)	5.2	2.8 - 9.7	<0.001
Anticoagulant used				
None	9/44 (20.5%)	(Reference)		
Heparin	7/51 (13.7%)	0.6	0.2-1.8	0.385
LMWH	38/207 (18.4%)	0.9	0.4 - 2	0.746
Bleeding complications				
No	44/270 (16.3%)	(Reference)		
Yes	10/31 (31.3%)	2.3	1 - 5.3	0.041
Insulin Infusion				
No	27/211 (12.8%)	(Reference)		
Yes	27/91 (29.7%)	2.9	1.6-5.3	<0.001
Furosemide use				
None	10/144 (6.9%)	(Reference)		
Bolus	22/94 (23.4%)	4.1	1.8 -9.1	<0.001
Infusion	22/64 (34.4%)	7	3.1 -16	<0.001
Acute Kidney Injury				
No	17/226 (7.5%)	(Reference)		
Yes	37/76 (48.7%)	11.7	6 - 22.8	<0.001

Table 4 summarises the distribution of the baseline characteristics of the cohort within the two intervention groups. The Control group participants were younger and less likely to have severe COVID-19 pneumonia than the Remdesivir group, both by clinical and radiological findings. However, certain comorbidities like chronic liver disease, chronic neurologic disease, and pregnancy were significantly more frequent in the control group.

**Table 4:** Distribution of demographic and clinical characteristics amongst Remdesivir vs Control groups (N =302) Distribution displayed using frequencies and column percentages, HRCT – High Resolution Computed Tomography, *n = 3 did not under go an HRCT.

Baseline characteristics	Remdesivir (n = 204 )	Control (n = 98 )	p-value
Age in years			
18-34	11 (5.4%)	15 (15.3%)	0.037
35-54	56 (27.5%)	25 (25.5%)	
55-64	57 (27.9%)	26 (26.5%)	
> 65	80 (29.2%)	32 (32.7%)	
Sex			
Male	161 (78.9%)	64 (65.3%)	0.016
Female	43 (21.1%)	34 (34.7%)	
Comorbidities			
Diabetes Mellitus	114 (55.9%)	47 (48%)	0.219
Hypertension	100 (49%)	51 (52%)	0.712
Chronic Liver Disease	3 (1.5%)	9 (9.2%)	0.003
Chronic Kidney Disease	20 (9.8%)	16 (16.3%)	0.128
Ischaemic Heart Disease	48 (23.5%)	30 (30.6%)	0.207
Chronic Lung Disease	18 (8.8%)	5 (5.1%)	0.355
Chronic Neurological Disease	2 (1%)	6 (6.1%)	0.016
Stroke	13 (6.4%)	4 (4.1%)	0.595
Pregnancy	4 (2%)	7(7.1%)	0.043
Cancer	14 (6.9%)	7 (7.1%)	1
Immunocompromised	2 (1%)	1(1%)	1
Morbidity count			
0-1	99 (48.5%)	40 (40.8%)	0.136
2-3	94 (46.1%)	47 (48%)	
> 3	11 (5.4%)	11 (11.2%)	
Admission Category			
Moderate	49 (24%)	65 (66.3%)	<0.001
Severe	155 (76%)	33 (33.7%)	
Admission HRCT Scoring			
Normal*	49 (24%)	71 (72.4%)	<0.001
Mild	11 (11.2%)	30 (14.7%)	
Moderate	52 (25.5%)	8 (8.2%)	
Severe	73 (35.8%)	8 (8.2%)	

Table 5 examines the crude associations of outcomes in detail. There was little to no evidence that Remdesivir was associated with all-cause mortality (cOR = 0.9, 95% CI = 0.5 -1.8, p = 0.878).

**Table 5:** Crude estimates associations of Remdesivir use and study outcomes (N=302). ECMO: Extracorporeal membrane oxygenation, SORT: Symptom Onset to Remdesivir Treatment, # - N = 229 patients who received at least one dose of Remdesivir, *Mean difference with standard error using an independent t-test.

Variables	Outcome/Total (%)	Crude Odds Ratio	95% CI	p-value
All-cause mortality				
Control	18/98 (18.4%)	(Reference)		
Remdesvir	36/204 (17.6%)	0.9	0.5 – 1.8	0.878
SORT interval & mortality#				
≤ 3 days	31/128 (24.2%)	(Reference)		
> 3 days	17/101 (16.8%)	0.6	0.3-1.2	0.165
Category escalation (Moderate to Severe)				
Control	16/98 (16.3%)	(Reference)		
Remdesivir	33/204 (16.2%)	0.9	0.5 – 1.9	0.974
Requirement of invasive ventilation				
Invasive Mechanical Ventilation/ECMO				
Control	14/98 (14.3%)	(Reference)		
Remdesivir	39/204 (19.1%)	1.4	0.7 – 2.8	0.303
Intensive Care unit stay in days				
Control	4 ± 5			
Remdesivir	9 ± 8	5 ± 1*	3 - 7	<0.001
Bleeding complications				
Control	7/98 (7.1%)	(Reference)		
Remdesivir	25/204 (12.3%)	0.9	0.5 -1.7	0.878
Acute Kidney Injury				
Control	21/98 (21.4%)	(Reference)		
Remdesivir	55/204 (27%)	1.8	0.7 – 4.3	0.182
Antibiotic escalation				
Control	19/98 (19.4%)	(Reference)		
Remdesivir	59/204 (28.9%)	1.7	0.9 - 3	0.078

Furthermore, there was no statistical evidence that Remdesivir use was associated with category escalation, escalation in the ventilatory requirement, bleeding complications, acute kidney injury, or antibiotic escalation. However, participants in the Remdesivir group were significantly more likely to spend 5 ± 1 more days in the ICU than the Control group. Table 6 depicts the co-interventions more likely to be used with Remdesivir rather than in the control group. In a subgroup analysis of participants in the Remdesivir group, there was no evidence of an association between SORT interval and mortality.

**Table 6:** Distribution of cointerventions amongst Remdesivir vs Control groups (N =302) HFNC: High Flow Nasal Cannula, BiPAP: Bilevel Positive Airway Pressure, ECMO: Extracorporeal membrane oxygenation, LMWH: Low Molecular Weight Heparin, # - Teicoplanin, Vancomycin, Clindamycin, Levofloxacin

Co-interventions	Remdesivir (n = 204 )	Control (n = 98 )	p-value
Initial mode of ventilation			<0.001
Room air	41 (20.1%)	54 (55.1%)	
Face mask	54 (26.5%)	6 (6.1%)	
Nasal prongs	52 (25.5%)	21 (21.4%)	
HFNC	2 (1%)	0	
BiPAP	42 (20.6%)	9 (9.2%)	
Invasive Mechanical Ventilation	13 (6.4%)	8 (8.2%)	
Steroid administration (0.5) (Interaction)			<0.001
None	11 (5.4%)	56 (57.1%)	
Bolus	118 (57.8%)	28 (28.6%)	
Infusion	75 (36.8%)	14 (14.3%)	
Steroid Dose			<0.001
None	10 (4.9%)	56 (57.1%)	
Methylprednisolone 40mg	56 (27.5%)	20 (20.4%)	
Methylprednisolone 80mg	138 (67.6%)	22 (22.4%)	
Antibiotics initiated			0.001
None	21 (10.3%)	28 (28.6%)	
Augmentin/Azithromycin	26 (12.7%)	11 (11.2%)	
Cefperazone	67 (32.8%)	26 (26.5%)	
Meropenem	27 (13.2%)	8 (8.2%)	
Piperacillin + Tazobactam	60 (29.4%)	21 (21.4%)	
Others#	3 (1.5%)	4 (4.1%)	
Anticoagulant used			<0.001
None	13 (6.4%)	31 (31.6%)	
Heparin	32 ( 15.7%)	19 (19.4%)	
LMWH	159 (77.9%)	48 (49%)	
Insulin Infusion			<0.001
No	126 (61.8%)	85 (86.7%)	
Yes	78 (38.2%)	13 (13.3%)	
Furosemide use			0.002
None	83 (40.7%)	61 (62.2%)	
Bolus	71 (34.8%)	23 (23.5%)	
Infusion	50 (24.5%)	14 (14.3%)	

While building the causal regression model, participant age and sex were detected as confounders (bivariate OR = 0.85 and 0.83, respectively) and were adjusted for in the final model. Furthermore, admission category and steroid use were detected to be significant effect modifiers. The final adjusted model showed that the crude estimate of association was attenuated slightly (aOR = 0.76, 95% CI = 0.4 - 1.5, p = 0.409). Nonetheless, it still did not provide evidence for an association between Remdesivir use and mortality.

However, when stratifying this adjusted estimate by the effect modifier "Admission Category", it was noted that in the "Severe" strata - Remdesivir is shown to have a strong negative association with all-cause mortality (aOR = 0.3, 95% CI = 0.1 - 0.6, p = 0.003). A similarly strong negative association between Remdesivir and all-cause mortality is seen in participants who received steroids as a co-intervention (Table 7).

**Table 7:** Final multivariate regression model and it's interaction terms (N = 302) *Adjusted for Age and Sex, ** From the likelihood-ratio test

Strata by effect modifier	Exposure of Interest	Death/Total (% died)	Adjusted Odds ratio*	95% CI	p-value
Unstratified/No effect modifier	Control	18/98 (18.4%)	(Reference)		
	Remdesvir	36/204 (17.6%)	0.76	0.4 – 1.5	0.409
Admission category					0.003**
Moderate	Control	3/65 (4.6%)	(Reference)		
	Remdesivir	7/49 (14.1)	2.8	0.7 - 11.8	
Severe	Control	15/33 (45.1%)	(Reference)		
	Remdesivir	29/155 (18.7%)	0.3	0.1 - 0.6	
Steroid use					0.065**
No	Control	2/56 (3.6%)	(Reference)		
	Remdesivir	2/11 (18.2%)	4.8	0.6 – 40.4	
Bolus	Control	9/28 (32.1%)	(Reference)		
	Remdesivir	17/118 (14.4%)	0.3	0.1 – 0.9	
Infusion	Control	7/14 (50%)	(Reference)		
	Remdesivir	17/75 (22.7%)	0.2	0.1 – 0.9	

## Discussion

This retrospective cohort study included patients with moderate to severe COVID-19 illness admitted for intensive care in the first wave of the pandemic in Kochi, India. At the time, there were no evidence-based guidelines for ICU management, and COVID-19 vaccinations were not yet available to the public. In our cohort, Remdesivir use was not associated with any improvement in all-cause mortality before hospital discharge or the requirement of invasive mechanical ventilation. The null association of Remdesivir with all-cause mortality persisted, even after being adjusted for confounders such as age and sex. However, when stratified for effect modifiers, Remdesivir seemed to be strongly associated with a decrease in all-cause mortality in patients with severe disease or in patients who received steroids as a co-intervention (either as a bolus dose or as an infusion).

Remdesivir may have had little to no effect on all-cause mortality in our cohort of intensive-care patients with COVID-19. Randomized controlled trials (RCT) have confirmed this null effect that recruited similar cohorts conducted in high and upper-middle-income countries [9]. A shorter SORT interval of fewer than three days did not confer a mortality benefit, contradicting previous studies [10,11]. However, our study design limits the validity of these interpretations. The probable presence of unmeasured potential confounders like Body Mass Index or residual confounding by the severity levels of comorbidities may have resulted in the estimates tending to null. Twenty-four participants in the "Control" group did not receive the standard five-day treatment of Remdesivir, as they developed a derangement in their liver functions or expired prior to the completion of the regimen. Since all-cause mortality was the primary outcome in the present study, the true effect of Remdesivir in preventing COVID-19 Acute Respiratory Distress Syndrome-associated deaths may have been obfuscated by other causes of death in the cohort. While RCTs suggest that Remdesivir use probably results in less requirement for mechanical ventilation in patients with severe COVID-19 or shorter recovery times in hospitalized patients (with less severe disease) [12,13], these effects were not reflected in our unadjusted estimates, probably due to confounding bias.

However, as a novel finding, our stratified estimates suggest that Remdesivir may be more protective against mortality in patients with severe COVID-19 illness or when administered with steroids. Disregarding the stratified odds ratios (which are exaggerated and imprecise - as the incidence of mortality is high in the small strata), the risk of mortality is more than halved by the use of Remdesivir in severely ill patients (18.7% vs. 45.1%) or when used along with Methylprednisolone (14.4% vs. 32.1% for bolus and 22.7% vs. 50% for infusions) when compared to the control group (Table 7). However, this mortality benefit of Remdesivir in severe COVID-19 illness has not been replicated in large RCTs [9,13]. The underlying pathophysiology of severe COVID-19 illness involves a late pulmonary phase dominated by alveolar damage - likely mediated by hyper-inflammatory processes, where a broad antiviral like Remdesivir probably has no role [9,14]. However, we hypothesize that the steroid use will suppress the hyper-inflammatory cascades, allowing Remdesivir to suppress any remaining viral activity, thus creating a synergistic effect for recovery. Recent observational studies may have detected a similar effect [15,16]. However, no RCTs have been conducted to test this hypothesis (to the best of our knowledge), which could help eliminate any unknown confounding. Our stratified estimates were too imprecise, as our study was not powered for stratified analysis and could be false positives - due to multiple tests.

Steroids like Dexamethasone [17,18] or Methylprednisolone [19] have shown substantial mortality benefits in severe COVID-19 illness. We recommend conducting a large, triple-blinded, parallel RCT comparing the mortality benefits of "Intravenous steroids plus Remdesivir" vs. "Intravenous steroids alone" that will test our derived hypothesis definitively. With the threat of an unexpected resurgence of the COVID-19 infection - anywhere in the world, possibly due to highly transmissible, hypermutable SARS-COV-2 variants and waning vaccine-mediated immunity, any intervention with a mortality benefit is invaluable. Despite the limitations, a broad-spectrum antiviral drug such as Remdesivir may be an effective co-intervention with steroid therapy in severely ill COVID-19 patients [20].

Strengths and limitations

The relatively modest sample size of ICU patients and confounder-adjusted effect estimates strengthen the present study. The retrospective, non-randomized, unblinded design and potentially unmeasured confounders limit the study's internal validity. As we included 12 patients who underwent partial treatment with Remdesivir (before cessation due to liver toxicity) in the Control group - we anticipate that this inclusion may have falsely tended the association to the null effect of Remdesivir. However, 8 out of 12 partially treated patients only received one dose, and three patients received two doses of Remdesivir before they developed suspected drug-induced liver injury and necessitated its cessation. It is unlikely that these patients gained any mortality benefit from the drug. As the number of partially treated patients in the control group is small and they received less than three doses - we believe that it may not have induced a false null association. The study was also conducted in a single center in India, limiting the external validity to similar tertiary/quaternary centers in the same population.

## Conclusions

In this retrospective analysis of moderate to severe adult COVID-19 ICU patients, Remdesivir was not found to be associated with a mortality benefit or other measured clinical outcomes. However, our stratified analysis suggests that Remdesivir may be associated with lower mortality rates in severely ill COVID-19 patients or when used alongside intravenous steroids. However, we could not establish causality due to the limitations of the present study and await larger RCTs that investigate the combined benefits of Remdesivir and steroids in severe COVID-19 illness to be conducted.
